# Combining *p*-values from various statistical methods for microbiome data

**DOI:** 10.3389/fmicb.2022.990870

**Published:** 2022-11-10

**Authors:** Hyeonjung Ham, Taesung Park

**Affiliations:** ^1^Interdisciplinary Program of Bioinformatics, Seoul National University, Seoul, South Korea; ^2^Departement of Statistics, Seoul National University, Seoul, South Korea

**Keywords:** microbiome analysis, integration method, *p*-value combination, power simulation, rank simulation

## Abstract

**Motivation:**

In the field of microbiome analysis, there exist various statistical methods that have been developed for identifying differentially expressed features, that account for the overdispersion and the high sparsity of microbiome data. However, due to the differences in statistical models or test formulations, it is quite often to have inconsistent significance results across statistical methods, that makes it difficult to determine the importance of microbiome taxa. Thus, it is practically important to have the integration of the result from all statistical methods to determine the importance of microbiome taxa. A standard meta-analysis is a powerful tool for integrative analysis and it provides a summary measure by combining *p*-values from various statistical methods. While there are many meta-analyses available, it is not easy to choose the best meta-analysis that is the most suitable for microbiome data.

**Results:**

In this study, we investigated which meta-analysis method most adequately represents the importance of microbiome taxa. We considered Fisher’s method, minimum value of *p* method, Simes method, Stouffer’s method, Kost method, and Cauchy combination test. Through simulation studies, we showed that Cauchy combination test provides the best combined value of *p* in the sense that it performed the best among the examined methods while controlling the type 1 error rates. Furthermore, it produced high rank similarity with the true ranks. Through the real data application of colorectal cancer microbiome data, we demonstrated that the most highly ranked microbiome taxa by Cauchy combination test have been reported to be associated with colorectal cancer.

## Introduction

Since the roles of the microbiome in human body sites and their importance arise, there have been many studies focusing on revealing differentially expressed microbiome taxa in a variety of cancer types and diseases (Hayes et al., 2018; Osman et al., 2018; Qian et al., 2018; Dong et al., 2019; Ramsheh et al., 2021). In the meanwhile, there are certain common characteristics among microbiome datasets that make analyses difficult: overdispersion and high sparsity (presence of zero counts; Sohn and Li, 2018; Xia et al., 2018). To account for these characteristics, many statistical methods have been developed. DESeq2 and edgeR are widely used methods to find differentially expressed features in the field of RNA-Seq data analysis, and account for overdispersion of the dataset using a negative binomial distribution modeling strategy (Robinson et al., 2010; Love et al., 2014). MetagenomeSeq was developed to account for sparsity using a distinct normalization method, known as cumulative sum scaling (CSS) and using a zero-inflated model (Paulson et al., 2013). ZIBseq and ZINB are methods that account for the sparsity through incorporating zero-inflated beta model and zero-inflated negative binomial model, respectively (Peng et al., 2016; Xia et al., 2018). There also are methods that use centered log-ratio (CLR) transformation to account for the compositional nature of relative abundance data in analysis (Gloor et al., 2017).

Microbiome analysis methods are broadly classified into two classes: taxa-level method and community-level method (Plantinga et al., 2017). Taxa-level method performs analyses in terms of each taxon, and includes aforementioned methods. The community-level method accounts for phylogenetic distances between representative sequences. MiRKAT, the microbiome regression-based kernel association test, uses kernels that incorporate microbiome-wise similarity matrix that can be calculated from various distances (Zhao et al., 2015). MiSPU, the microbiome-based sum of powered score, uses the idea of the sum of powered score (SPU) to be applied to microbiome datasets through variable weighting of representative sequences (Wu et al., 2016). OMiAT, optimal microbiome-based association test, is an approach that integrates SPU and MiRKAT by taking the minimum value of *p* from the two methods (Koh et al., 2017). TMAT, the phylogenetic tree-based microbiome association test, uses log-transformed read count per million (CPM) and tests whether an internal node of a phylogenetic tree is associated with the outcome, using the phylogenetic tree structure (Kim K. J. et al., 2020). All the methods introduced above are used to find the differentially expressed (DE) features. There have been studies that attempted a comprehensive review of these statistical methods (Xia and Sun, 2017; Pollock et al., 2018; Nearing et al., 2022). However, it is not easy to tell which is the best method among the individual DE method because each method is specialized for the specific characteristics of microbiome data. Furthermore, the significance results provided from different statistical methods tend to be inconsistent. In other words, a DE feature from one method does not necessarily be a DE feature from the other method (Khomich et al., 2021). Thus, several studies summarized the inconsistent results obtained from different statistical methods by using a Venn diagram that represented commonly significant features under a certain significance level (Chen et al., 2015; You et al., 2018; Nazarieh et al., 2019; Wang et al., 2019; Kim S. I. et al., 2020). In addition to the significance, the ranking of DE features is also inconsistent between the methods.

In this study, we combine the value of *p*s from different statistical methods to determine the importance of DE features. Rather than focusing on an individual method, our focus lies in combining different test results from a set of multiple methods. There exist many methods for combining value of *p*s, depending on whether value of *p*s are independent (Fisher, minimum value of *p*, Simes, Stouffer) or correlated (Kost, Cauchy). The most common method is Fisher’s method that uses a chi-square distribution to calculate the combined value of *p* (Fisher, 1925). The method using the minimum value of *p* can also be taken to maximize the power (Tippett, 1931; Casella and Berger, 2017). Simes method for combining value of *p* is similar to the minimum value of *p* method, but uses ordered value of *p*s to determine the significance (Simes, 1986). Stouffer’s method takes the inverse standard normal cumulative distribution function (CDF) of value of *p*s so that the statistic follows a normal distribution (Stouffer and Suchman, 1949). Kost method accounts for the correlation between *p*-values by modifying the chi-square distribution of the Fisher’s method (Kost and McDermott, 2002). Cauchy combination test accounts for the correlation between *p*-values by using Cauchy distribution, which makes the distributional changes in the tail limited in the existence of *p*-value correlation (Liu and Xie, 2020). The combined *p*-values were then used to rank the importance of microbiome.

In this study, we investigate the most appropriate *p*-value combination method in the analysis of microbiome dataset in terms of significance testing and ranking DE features. Simulation settings were designed to assess: (i) the type 1 error and power of differentially expressed feature discovery, (ii) rank similarity between the true ranks and ranks determined by combined *p*-values.

In our empirical studies, we only considered the genus level. Many differential abundance analyses have been conducted only at the genus level, due to the limitation in microbiome annotation and not enough high resolution provided by 16 s rRNA sequence to classify species. Popular microbiome databases, including Silva, and Greengenes databases, recommend not to use the annotation at the species level (Ritari et al., 2015; Dueholm et al., 2020). Although databases such as NCBI and EzBioCloud EzTaxon provide more accurate annotations than Silva and Greengenes at the species level (Kim et al., 2012; Schoch et al., 2020), uncultured and unidentified species still exist and are often filtered out in the differential abundance analyses. Additionally, the microbiome resolution provided by 16 s rRNA is limited because the length of highly variable region is short for accurately classifying species except for few species. Therefore, analysis was conducted in the genus level at this study.

## Materials and methods

### Microbiome datasets

#### Baxter’s colorectal cancer data

Stool samples obtained through the Great Lakes-New England Early Detection Research Network were used in this study (Baxter et al., 2016). Raw sequencing data and metadata are available at NCBI Sequencing Read Archive (SRA) with the accession number SRP062005. A total of 314 samples with 187 normal and 127 colorectal cancer (CRC) were available.

Experimental procedures were previously reported as follows (Kozich et al., 2013). The V4 region of 16 s rRNA gene was amplified using custom-designed primers, and sequenced using an Illumina MiSeq sequencer with paired-end sequencing. Raw FASTQ data were processed through Qiime2 pipeline from raw file processing to taxonomy assignment (https://qiime2.org/, version 2021.04). Qiime2 Cutadapt plugin was used to trim primer sequences, and representative sequences were obtained through DADA2 denoising algorithm. Taxonomies were assigned using SILVA databases (release 138) with 99% similarity. Fasttree plugin was used to generate the phylogenetic tree. After removing singletons and doublets, data comprised 4,772 representative sequences. After filtering representative sequences with <0.005% of total read count (Bokulich et al., 2013), 803 representative sequences with 80 genera were available.

#### Zeller’s colorectal cancer data

Stool samples obtained through the European Molecular Biology Laboratory (EMBL) were used in the real analysis of this study. Raw sequencing data and metadata are available at European Nucleotide Archive (ENA) with the project number PRJEB6070. Excluding samples without the disease status information, a total of 91 samples with 50 normal and 41 CRC were available.

Experimental procedures were previously reported as follows (Zeller et al., 2014). The V4 region of 16 s rRNA gene was amplified using targeted primers (F515 5’-GTGCCAGCMGCCGCGGTAA-3′, R806 5’-GGACTACHVGGGTWTCTAAT-3′), and sequenced following Illumina MiSeq platform (Illumina, San Diego, United States) at the Genomics Core Facility, EMBL, Heidelberg. Raw FASTQ data were processed through the same pipeline as the Baxter’s data described above using Qiime2. After the filtering, 329 representative sequences with 81 genera were available.

### Methods for identifying DE features

The methods for identifying DE features are classified into taxa-level and community level methods, as summarized in Table 1 with the corresponding null hypotheses. Taxa-level method includes DESeq2[Wald/LRT], edgeR, Wilcoxon rank sum test with CLR transformation (Wilcoxon CLR), ZIBSeq, MetagenomeSeq [Gaussian/log normal], and ZINB. Community-level method includes oMiRKAT, aMiSPU, aSPU, and TMAT. aSPU was considered instead of OMiAT, that takes the minimum value of *p* of SPU and MiRKAT. For this study, the value of *p* generated by MiRKAT was already included, so only value of *p* generated by SPU was considered. All analysis results were obtained at the genus level. R^1^ software was used for the analyses. Unless stated, default options were used for all analysis.

**Table 1:** Null hypotheses of statistical methods.

Category	Method	Null hypothesis (H0)	Detail
Taxa-level	DESeq2	$\beta_{i}=0$	β =LFC (Log-fold change) For $i^{\mathrm{th}}$ taxa
	edgeR	$\lambda_{1}-\lambda_{2}=0$	For group 1, group 2
	Wilcoxon CLR	$\lambda_{1}^{median}-\lambda_{2}^{median}=0$	For group 1, group 2
	ZIBSeq	$\beta_{i}=0$	For $i^{\mathrm{th}}$ taxa
	MetagenomeSeq	$\beta_{i}=0$	For $i^{\mathrm{th}}$ taxa
	ZINB	$\beta_{i}=0$	For $i^{\mathrm{th}}$ taxa
Community-level	oMiRKAT	τ=0	Kernel regression Random effect *f*(*Z*) ~ (0, τ K) For kernel K $f\left(Z_{i}\right)=\overset{p}{\underset{j=1}{\sum }}Z_{ij}\beta_{j}$ , if the model is linear for p OTUs
	aMiSPU	$\beta ={\left(\beta_{1},\beta_{2},\ldots ,\beta_{p}\right)}^{\prime }=0$	For p OTUs
	aSPU	$\beta ={\left(\beta_{1},\beta_{2},\ldots ,\beta_{p}\right)}^{\prime }=0$	For p OTUs
	TMAT	$\beta ={\left(\beta_{1},\beta_{2},\ldots ,\beta_{M\_1}\right)}^{\prime }=0$	For M_1 internal nodes in phylogenetic tree

### Methods for integration analysis

For the value of *p* combination, Fisher’s method, minimum value of *p* method (min P method), Kost method, Simes method, Stouffer’s method, and Cauchy combination test were used. Details of each method are described below.

#### Fisher’s method

It is also called Fisher’s combination test. Under the null hypothesis, for independent value of ps,


$$T_{\mathrm{Fisher}}=-\overset{k}{\underset{i=1}{\sum }}2\log p_{i}\sim \chi_{2k}^{2}$$

for k tests to be combined, and $p_{i}$ represents $i^{\mathrm{th}}$ value of *p*.Minimum value of *p* method (Min P method). Under the null hypothesis, for independent value of *p*s,


$$T_{\min P}=\underset{i=1,2,\ldots ,k}{\min}p_{i}\sim \mathrm{Beta}\left(1,k\right)$$

for k tests to be combined.

#### Kost method

For dependent value of *p*s, scale the chi-square distribution of Fisher’s method as follows (Kost and McDermott, 2002):


$$T_{\mathrm{Kost}}\sim c\chi_{2f}^{2}$$

where


$$f=\frac{E{\left[T\right]}^{2}}{\mathrm{var}\left[T\right]},c=\frac{\mathrm{var}\left[T\right]}{2E\left[T\right]}=\frac{k}{f}$$

and


$$E\left[T\right]=2k,\mathrm{var}\left[T\right]=4k=2\underset{i<j}{\sum }\mathrm{cov}\left(-2\log p_{i},-2\log p_{j}\right)$$

#### Cauchy combination method

For value of *p*s under arbitrary dependency structure, defined by the weighted sum of the Cauchy transformed value of each value of *p* as follows:


$$T_{\mathrm{Cauchy}}=\overset{k}{\underset{i=1}{\sum }}w_{i}\tan\{\left(0.5-p_{i}\right)\pi )\}$$

where $w_{i}$ is nonnegative weight that satisfies $\sum_{i=1}^{k}\;\omega_{i}=1,$ and $p_{i}$ is the value of *p* from i th test. Cauchy combination test accounts for the dependence of value of *p*s using the heaviness of the Cauchy tail (Liu and Xie, 2020). Equal weights were used in this study.

#### Simes method

For independent value of *p*s, let $p_{1},\ldots ,p_{k}$ be the ordered *p*-values for k tests. The null hypothesis is rejected if $p_{i}\leq i\alpha /k$ for any i=1,…,k for a significance level α . It is mainly used in multiple testing correction, but also suggested for the *p*-value combination in some studies (Cheng and Sheng, 2017; Ganju and Ma, 2017).

#### Stouffer method

For independent *p*-values,


$$T_{\mathrm{stouffer}}=\frac{\sum_{i=1}^{k}\Phi^{-1}\left(1-p_{i}\right)}{\sqrt{k}}\sim N\left(0,1\right)$$

where Φ represents the standard normal cumulative distribution function.

### Simulation settings

#### Simulation setting 1

Simulation setting 1 was designed to assess type 1 error rates and power of each *p*-value combination method. The simulation datasets were generated as previously reported (Zhao et al., 2015). Microbiome datasets were simulated according to Chen and Li’s approach (Chen and Li, 2013). The simulated OTU counts were generated using Dirichlet-multinomial (DM) model, that incorporates the mean OTU proportion and the overdispersion measure as the shape parameter *α*. The sample size was set to 300 and 20,000 total read counts were generated per sample. The OTU counts were set to have different levels of sparsity (e.g., the total proportion of zero counts) to account for the zero-inflated nature of microbiome datasets. For sparsity, sparsity parameter π ϵ {0.3,0.5,0.7,0.8} was set. The OTU counts were simulated as follows:


$$Z_{ij}=\left\{\begin{array}{c} 0\;\mathrm{with probability}\;\pi \\ \mathrm{Dirichlet}-\mathrm{multinomial}\left(\alpha \right)\;\mathrm{with probability}\;1-\pi \end{array}\right.$$

where $Z_{ij}$ is OTU counts for ith sample and jth OTU.

The dependent variable was generated as practiced in MiRKAT (Zhao et al., 2015). For the binary outcome variable, the outcome was simulated under the model


$$\mathrm{logit}\left(E(y_{i}\mathrm{|,}X_{i}\mathrm{|,}Z_{i}\right))=0.5\mathrm{scale}\left(X_{1i}+X_{2i}\right)+\beta \mathrm{scale}\;\left(\underset{j\in G}{\sum }Z_{ij}\;\right)$$

where $y_{i}$ represents the dependent variable of the sample i , $X_{i}$ represents the covariates of sample i , scale( · ) represents the standardization with mean 0 and standard deviation 1, β represents the degree of association and G represents the given cluster of OTUs. Here, the OTU-level datasets are simulated so that each cluster of OTUs indicates each genus. Among the statistical methods, the taxa level analysis methods used a collapsed sum of OTUs corresponding to a genus, while the community-level analysis methods used simulated OTU data as it is.

One virtual covariate $X_{1i}$ was simulated as ~Bernouill(0.5) . The other virtual covariate $X_{2i}$ was simulated as ~N(0,1) , assuming the covariate and the taxa counts $Z_{i}$ were independent. β was set to have the values of {0,0.01,0.02,0.03,0.04,0.05,0.1,0.15,0.2} . Type 1 error was measured when β=0 . A total of 1,000 dependent variables were generated for each combination of β s and π s to calculate the type 1 error rates and the power.

Among the DE feature analysis methods, the taxa-level analysis methods used a collapsed sum of OTUs corresponding to a genus, while the community-level analysis methods used simulated OTU data as it is.

#### Simulation setting 2

**Figure 1:**
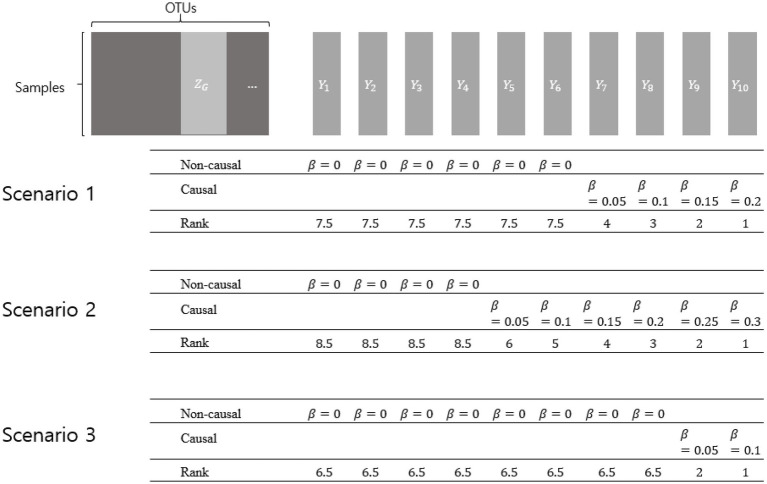
Simulation setting 2 with different scenarios. $Z_{G}$ represents a collection of OTUs comprising a genus. Ys represent dependent variables that were generated by various effect sizes (*β*s). The larger effect size represents the higher rank. Non-causal *β*s have the value of zero, while causal βs have the value of more than zero.

Simulation setting 2 was designed to assess the rank similarity between the true rank and the rank determined using each value of *p* combination method. The real CRC dataset introduced in Method 2.1 was used to reflect the microbiome counts of the real data. To control the degree of association ( β ), the dependent variable was generated under the same model from simulation setting 1. For the same dataset, ten different dependent variables were generated by previously determined β s as in Figure 1. The larger effect size, the higher rank. For each dependent variable, 100 replications were performed.

Three scenarios were considered using different numbers of non-causal dependent variables. Non-causal dependent variables were set to have β =0, assuming microbiome features that are not related to the dependent variables. Each scenario was designed to, respectively, have 6, 4, and 8 non-causal dependent variables, and the causal dependent variables were generated to have different degrees of association (with different *β*s).

The rank difference was presented with two measurements: rank squared difference and Spearman correlation coefficient. The rank squared difference was measured using


$$\overset{N}{\underset{g=1}{\sum }}d_{g}^{2}$$

where $d_{g}=\left(\mathrm{real}\;rank-\mathrm{computed}\;rank\right)$ of g th genus.

Similarly, the Spearman rank correlation coefficient was used as:


$$\rho =1-\frac{6\sum d_{g}^{2}}{N\left(N^{2}-1\right)}$$

With each value of *p* combination method, both measures were applied and the results of 100 replications were compared.

## Results

### Simulation result

#### Result of simulation setting 1

In this section, the type 1 error rates and the power of individual statistical method are first shown, then that of value of *p* combination methods are subsequently shown.

The type 1 error rates of individual methods are given in Table 2. Under the significance level of 0.05, the type 1 error rates of most statistical methods were well-controlled below 0.05. The type 1 error rates of ZIG Gaussian was uncontrolled in some cases, but not in ZIG log Normal. It was previously reported that the type 1 error rate of ZIG Gaussian was off the nominal range, compared to other statistical methods (Calgaro et al., 2020).

**Table 2:** Type 1 error rates of individual statistical methods.

Sparsity	DESeq2 LRT	DESeq2 Wald	edgeR	Wilcoxon	ZIBSeq	ZIG Gaussian	ZIG Log Normal	ZINB	aSPU	oMiRKAT	aMiSPU	TMAT
0.3	0.000	0.000	0.000	0.017	0.002	0.000	0.000	0.000	0.014	0.014	0.014	0.014
0.5	0.037	0.037	0.026	0.006	0.031	**0.094**	0.000	0.043	0.042	0.042	0.042	0.042
0.7	0.000	0.000	0.000	0.013	0.000	0.009	0.000	0.000	0.000	0.000	0.000	0.000
0.8	0.000	0.000	0.000	0.000	0.000	0.000	0.000	0.000	0.000	0.000	0.000	0.000

Bold value indicates to inflated type I errors.

**Figure 2:**
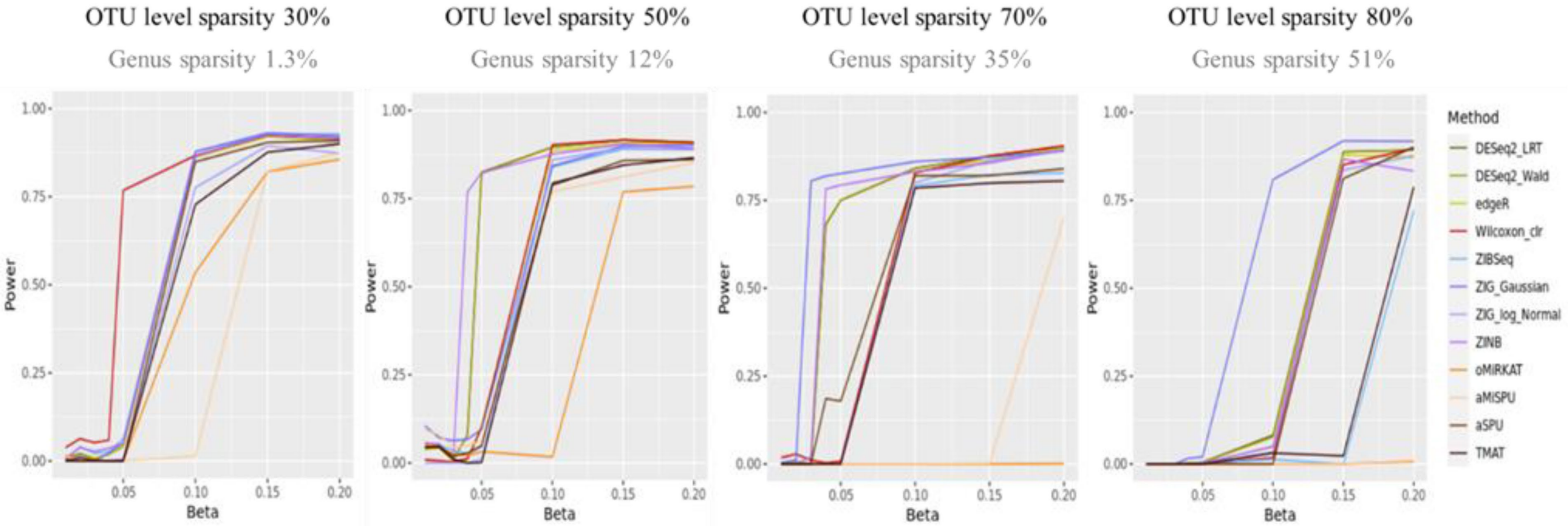
The statistical power of the individual methods. The *x*-axis represents the degree of association ( β ). The value of βs were given as {0.01,0.02,0.03,0.04,0.05,0.1,0.15,0.2} . The y-axis represents the power. The blueish colors represent methods that consider zero-inflation.

Figure 2 shows the statistical power of the individual methods in terms of the degree of association. The power tended to decrease as the level of sparsity increased, and the power of community-level analysis methods tended to be lower than the taxa-level analysis methods. The methods used in RNA-Seq data analysis showed higher performances in terms of power (ZIG, DESeq2). The Wilcoxon rank sum method showed a higher performance when the sparsity level was low (Genus sparsity 1.3%).

Table 3 represents the type 1 error rates of value of *p* combination methods. The type 1 error rates were not controlled in Fisher’s method and Stouffer’s method. The type 1 error rates were considered to be not controlled if the confidence interval for proportion test did not include 0.05 (i.e., for Stouffer’s method with sparsity 0.3, the 95% confidence interval of [0.0694, 0.1051] did not include 0.05, for Cauchy combination test with sparsity 0.5, the 95% confidence interval of [0.0435, 0.0732] include 0.05.). The type 1 error rates of other value of p combination methods did not exceed the given significance level of 0.05 considering the confidence interval. Since the type 1 error rates of Fisher’s combination method and Stouffer’s methods were not controlled, we focused only on the other methods for value of *p* combination. The results for Fisher’s and Stouffer’s methods can be found in the Supplementary Figure 1.

**Table 3:** The type 1 error rates of p-value combination methods.

Sparsity	Fisher	MinP	Kost	Cauchy	Simes	Stouffer
0.3	0.032	0	0.005	0	0	**0.086**
0.5	**0.09**	0.029	0.045	0.057	0.032	**0.096**
0.7	0.01	0	0	0	0	0.016
0.8	0	0	0	0	0	0.005

Bold value indicates to inflated type I errors.

**Figure 3:**
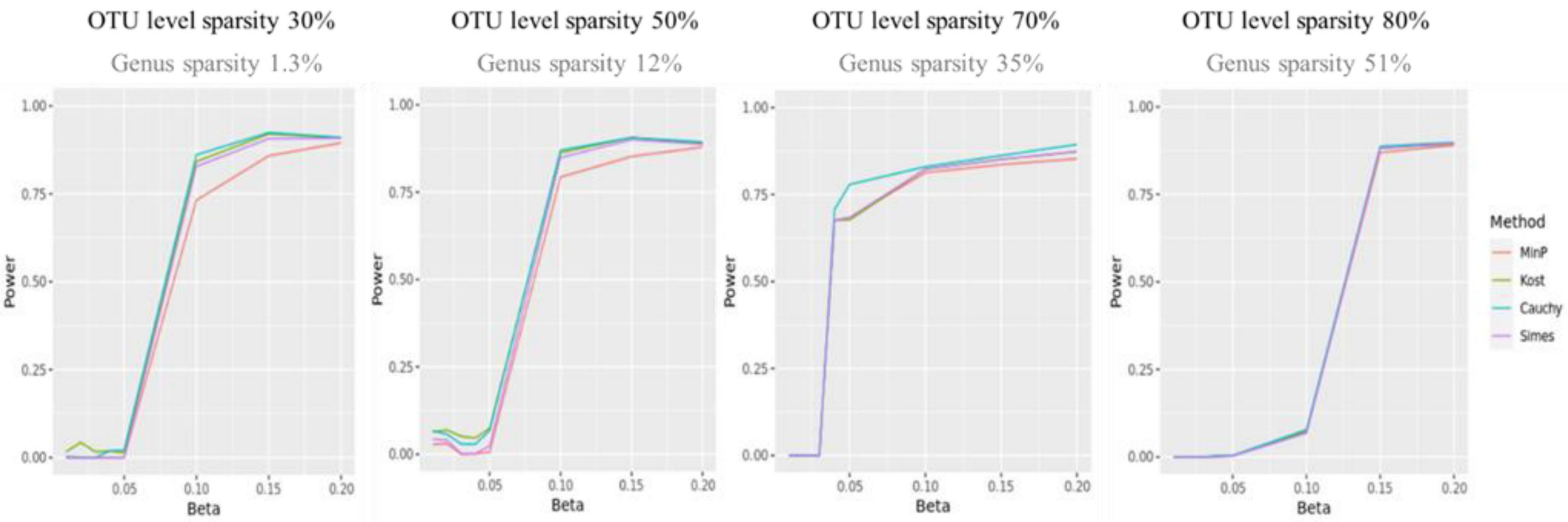
The statistical power of value of *p* combination methods. The *x*-axis represents the degree of association ( β ). The value of βs were given as {0.01,0.02,0.03,0.04,0.05,0.1,0.15,0.2} . The *y*-axis represents the power.

Figure 3 shows the statistical power of the value of *p* combination methods as the degree of association increases. Although the performances of value of *p* combination methods were similar, the power of Cauchy combination test was observed to be the best for all levels of sparsity. The performance of min *P* method was the worst. The differences in power between the methods tended to be smaller as the sparsity level becomes higher.

#### Result of simulation setting 2

**Figure 4:**
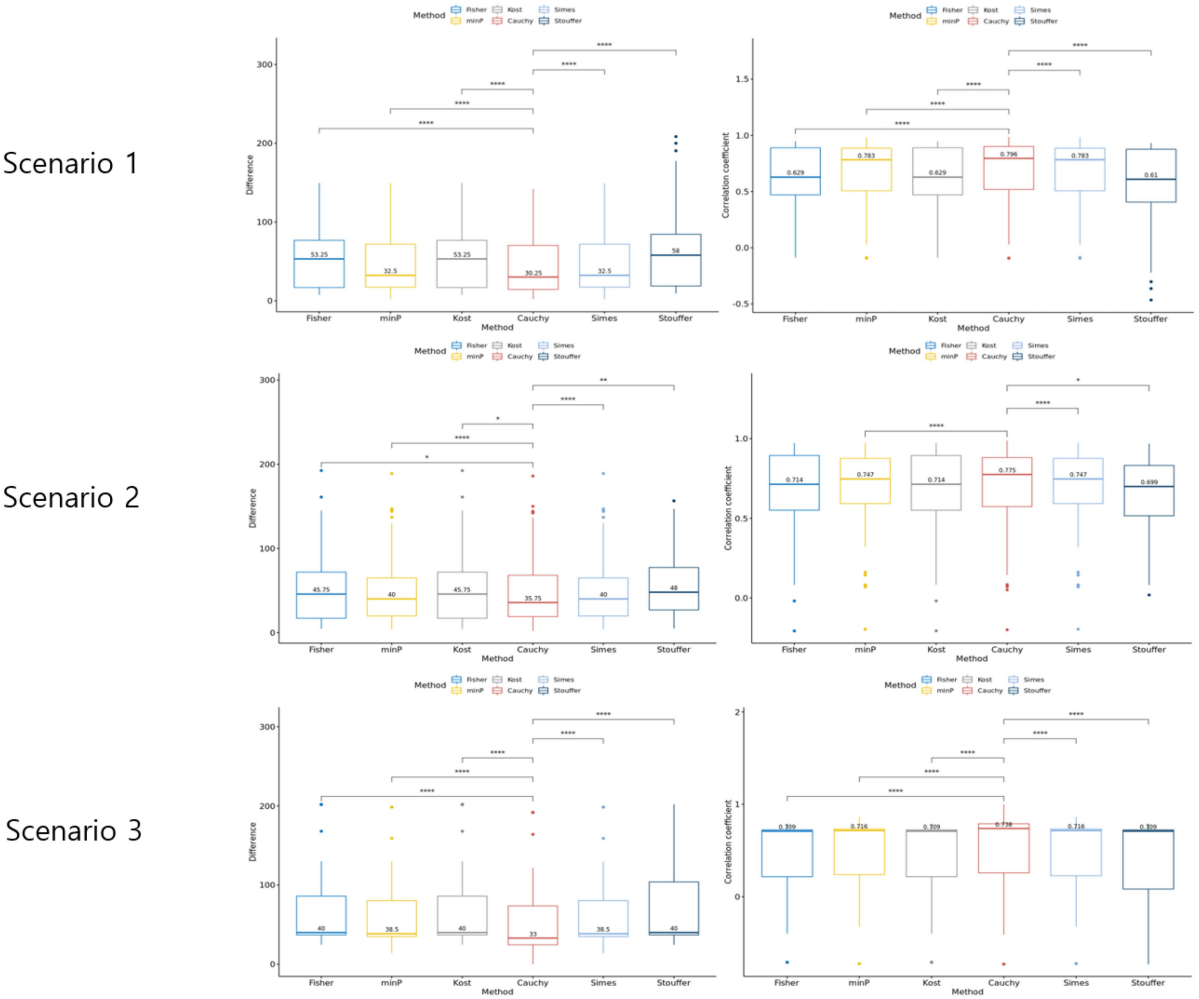
Results of the simulation setting 2. The graphs in the left column represent rank difference of each value of *p* combination method. The graphs in the right column represent the Spearman rank correlation of each *p*-value combination method.

Three scenarios were considered to evaluate the rank difference. In scenario 1, the rank squared difference was the lowest when combined with Cauchy combination test, Min P and Simes methods being next (Figure 4). Similarly, the Spearman rank correlation was the highest for Cauchy combination test. In both measures, the paired Wilcoxon test value of *p* between Cauchy combination test results and others were significant (value of *p* < 0.001). Similarly, Cauchy combination test showed the lowest rank squared difference and the highest correlation coefficient in scenarios 2 and 3 (Figure 4).

### Real microbiome data analysis

#### Baxter’s colorectal cancer data analysis

**Figure 5:**
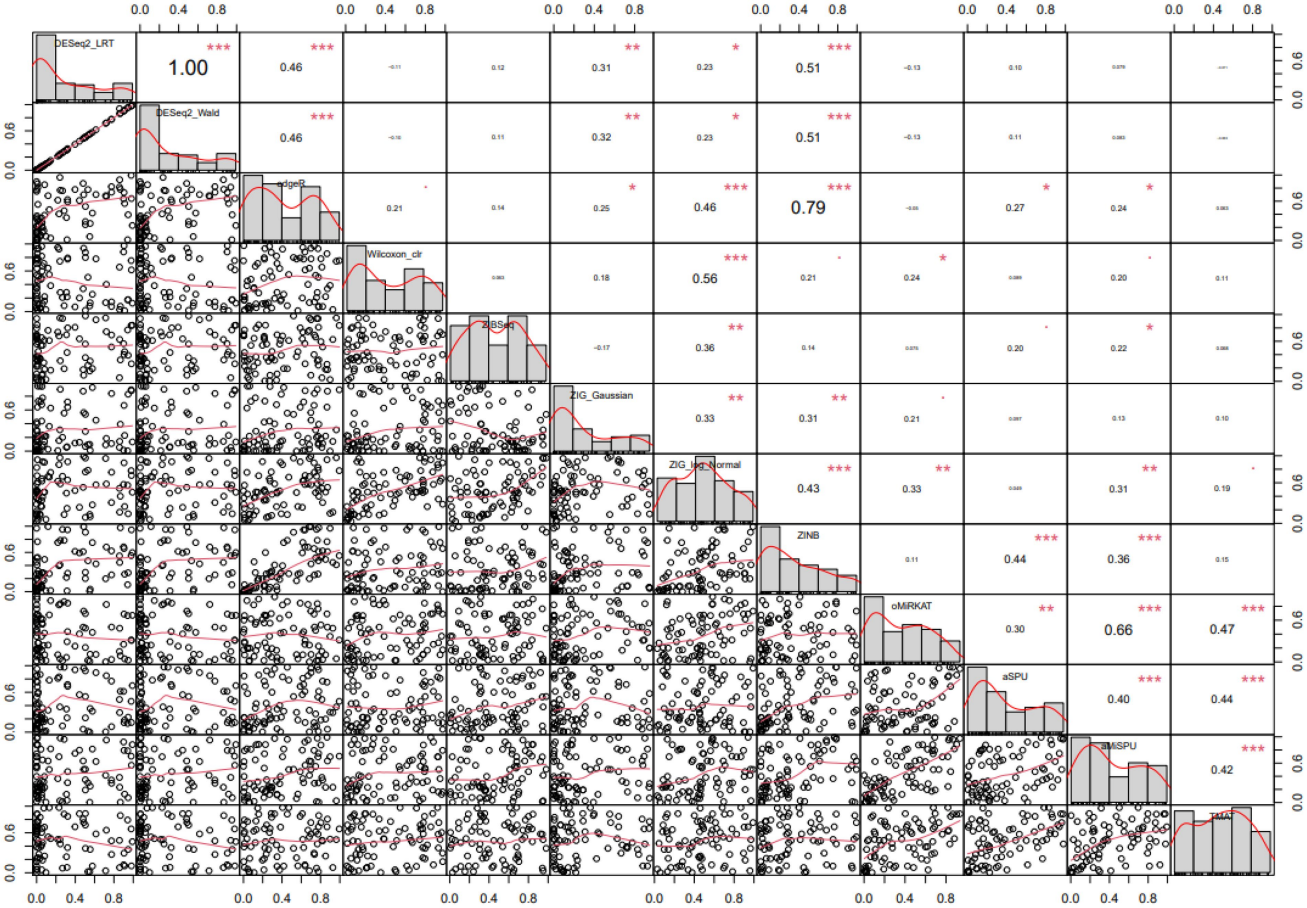
Pairwise Spearman correlation coefficients computed from various statistical methods. Spearman correlation of each pair of methods are represented in the upper diagonal graphs. The bigger the numbers the stronger the correlation. Lower diagonal scatterplots represent *p*-values. Diagonal graphs with the method name have histogram of p-values. Methods are in the order of DESeq2[LRT/Wald], edgeR, Wilcoxon CLR, ZIBSeq, ZIG[Gaussian/log normal], ZINB, oMiRKAT, aSPU, aMiSPU, TMAT.

The differentially expressed microbiome feature analysis was conducted for every genus in the Baxter’s CRC dataset, and the importance was determined by the magnitude of value of *p*s generated for each genus. DE feature analyses were used as described in the Method section. The Spearman rank correlation between each pair of statistical methods was compared as in Figure 5. A Spearman rank correlation coefficient of 0.46 was observed between DESeq2 and edgeR, which are both used in RNA-Seq analysis and based on the negative binomial distribution in common. A lower spearman rank correlation coefficient was observed between edgeR and Wilcoxon rank sum test results, between ZIBSeq and others, ZIG and others, ZINB and others except for RNA-Seq analysis methods, and the community-level analysis methods (oMiRKAT, aSPU, aMISPU, and TMAT) and others. The correlation tests were significant between some pairs of methods, that means there was a linear trend between value of *p*s ranks generated for those methods. However, the linear trend does not assure that the pairwise *p*-values have the same ranks. For example, although the correlation test between edgeR and ZINB is significant with the coefficient of 0.79, and thus they have a linear trend of *p*-value ranks, the pairwise *p*-values are not aligned as DESeq2_LRT and DESeq2_Wald. Furthermore, except for the DESeq2_LRT and DESeq2_Wald, which are both derived from DESeq2, no pair of methods produced similar rank list of microbiome genera (Supplementary Figure 2).

CRC stool samples were analyzed with different statistical methods and the resulting *p*-values were combined using Cauchy combination test. These *p*-values were further adjusted for controlling the false discovery rate (FDR) as practiced (Yoon et al., 2021). Table 4 shows the top microbiome genera in the order of adjusted *p*-values (*q*-values).

**Table 4:** Top 10 microbiome genera ranked by Cauchy combination test.

Taxa (o:order, f:family, g:genus)	*q*-value
*o__Rhodospirillales; f__uncultured; g__uncultured*	5.64E-20
*o__Veillonellales-Selenomonadales; f__Veillonellaceae; g__Megasphaera*	1.22E-16
*o__Gastranaerophilales; f__Gastranaerophilales; g__Gastranaerophilales*	3.72E-15
*o__Synergistales; f__Synergistaceae; g__Cloacibacillus* *	7.42E-13
*o__Bacteroidales; f__Porphyromonadaceae; g__Porphyromonas* *	4.23E-09
*o__Clostridia_vadinBB60_group; f__Clostridia_vadinBB60_group; g__Clostridia_vadinBB60_group* *	1.12E-07
*o__Burkholderiales; f__Sutterellaceae; g__Sutterella*	2.13E-05
*o__Bacteroidales; f__Marinifilaceae; g__Odoribacter*	1.09E-05
*o__Erysipelotrichales; f__Erysipelotrichaceae; g__Turicibacter*	1.51E-04
*o__Coriobacteriales; f__Eggerthellaceae; g__Slackia*	1.87E-04

* Commonly significant microbiome genera with Zeller’s data.

The first taxon was the most significant. Although it was uncultured in both genus and family levels, *Rhodospirillales* in order level was previously identified in the dextran sulfate sodium-induced colitis group but not in the control group (Yang et al., 2017). Also, the microbiome family *Rhodospirillaceae* was increased in colitic mice and IBD patients (Burrello et al., 2018). The bacterial genus *Megasphaera* was found to be a butyrate-producer, that induces epigenetic modifications in CRC development (Tarashi et al., 2019). *Gastranaerophilales* was previously reported as correlated with the late phase of aging through gene expression profiles of C57BL/6 J mice (van der Lugt et al., 2018). The genus *Cloacibacillus* was observed to be enriched in CRC patients with stage IV (Sheng et al., 2019). The bacterial species *Porphyromonas asaccharolytica* and *Porphyromonas gingivalis*, both rarely detectable in healthy individuals, were shown to be enriched in CRC patients in previous studies (Sinha et al., 2016; Okumura et al., 2021; Wang et al., 2021). Clostridia vadinBB60 group was observed to be enriched in low-graded; right-sided/transverse tumors (Zwinsová et al., 2021). The genus *Sutterella* was reported to be the most representative in the colorectal adenocarcinoma groups (Mori et al., 2018). The bacterial species *Odoribacter splanchnicus* was previously reported as a potential inducer of TH17cells and might protect against colitis and CRC in wild type mice (Xing et al., 2021; York, 2021). The abundance of *Turicibacter* was observed to be higher in the colitis or CRC group than in the groups with treatments, but the causative role of *Turicibacter* is to be further studied (Wu M. et al., 2019). The genus *Slackia* was studied to be overrepresented in CRC (Coleman and Nunes, 2016).

Most microbiome genera in Table 4 that had high ranks from Cauchy combination test had been previously reported as associated with CRC or related symptoms. The ranks generated by min P and Simes method were similar to the Cauchy combination test, which corresponds to the results from the simulation setting 2. On the other hand, the other methods did not include some highly ranked taxa discovered from Cauchy combination test in the lists of their top 10 taxa (Supplementary Table 1). For example, Cauchy combination test ranked the genera *Sutterella* and *Odoribacter* at 7^th^ and 8^th^, while Stouffer’s method ranked them at 18^th^ and 13^th^, respectively, despite their reported associations with CRC.

#### Zeller’s colorectal cancer data analysis

A different CRC stool samples were analyzed with statistical methods and the resulting *p*-values were combined using Cauchy combination test. Table 5 shows the top microbiome genera in the order of q-values.

**Table 5:** Top 10 microbiome genera ranked by Cauchy combination test.

Taxa (o:order, f:family, g:genus)	*q*-value
*o__Bacteroidales; f__Porphyromonadaceae; g__Porphyromonas* *	1.80E-14
*o__Lachnospirales; f__Lachnospiraceae; g__Hungatella*	3.26E-13
*o__Fusobacteriales; f__Fusobacteriaceae; g__Fusobacterium*	2.78E-09
*o__Bacteroidales; f__Rikenellaceae; g__Rikenellaceae_RC9_gut_group*	1.11E-06
*o__Synergistales; f__Synergistaceae; g__Cloacibacillus* *	1.35E-06
*o__Veillonellales-Selenomonadales; f__Veillonellaceae; g__Veillonella*	1.47E-06
*o__Erysipelotrichales; f__Erysipelatoclostridiaceae; g__Catenibacterium*	1.11E-05
*o__Veillonellales-Selenomonadales; f__Selenomonadaceae; g__Mitsuokella*	1.36E-05
*o__Desulfovibrionales; f__Desulfovibrionaceae; g__Bilophila*	5.75E-05
*o__Lachnospirales; f__Lachnospiraceae; g__Anaerostipes*	1.05E-04

* Commonly significant microbiome genera with Baxter’s data.

The most significant microbiome, *Porphyromonas* has been reported to be enriched in gut microbiota profiling of CRC patients in several studies (Yang et al., 2019). *Hungatella* was found to be a CRC-enriched marker, and was found to be depleted after the removal of CRC compared with newly diagnosed CRC patients (Cronin et al., 2022). Also, the species *Hungatella hathewayi WAL-18680* is a common cancer-associated biomarker (Wu et al., 2021). *Fusobacterium nucleatum* is commonly associated with CRC, and found to promote tumor development by inducing several immune responses including inflammation (Wu J. et al., 2019; Queen et al., 2022). *Rikenellaceae RC9 gut group* was suggested as a potential biomarker of CRC from gut microbiota profiles in mice (Shao et al., 2022). *Cloacibacillus* was reported to show statistical differences in the gut microbiota between CRC patients with stage III and IV (Sheng et al., 2019). *Veillonella* and a strain of *Streptococcus* together were reported to modulate inflammation, and were increased in fibrosis and cirrhosis compared to samples without cirrhosis (Jia et al., 2021). The relative abundance of *Catenibacterium* was found to be significantly different between CRC and normal patients (Yang et al., 2019). A low abundance of *Mitsuokella* in CRC patients compared to healthy controls was reported (Sobhani et al., 2019). *Bilophila wadsworthia* was reported to produce genotoxic hydrogen sulfide in the gut, enhancing carcinogenesis (Coker et al., 2022). The relative abundance of *Anaerostipes* were reported to be reduced in CRC patients compared to healthy controls (Chen et al., 2012).

Similar to the previous results with Baxter’s data, Fisher’s method, Kost’s method, and Stouffer’s method ranked CRC-related important genera lower than Cauchy combination test. For example, *Fusobacterium*, which was ranked 3^rd^ by Cauchy combination test, was ranked 12th, 12th, and 36th, respectively. Similarly, *Cloacibacillus*, which was ranked 5^th^ by Cauchy combination test, was ranked 15.5th, 15th, and 18th, respectively.

We also compared the results obtained from two CRC datasets (Baxter’s data and Zeller’s data). A total of 64 common genera were found. *Fusobacterium* was found to be the rank of 27.5 out of 80 genera in Baxter’s data, but the rank of 3 out of 81 genera in Zeller’s data. The value of *p* trend of the two datasets, and there were four commonly significant genera (*q*-value <0.05). *Fusobacterium* was found to be significant in Zeller’s data, but not in Baxter’s data with *q*-value of 0.133.

The commonly significant genera from the real datasets were investigated. There were 22 significant microbiome genera (*q*-value <0.05) from Zeller’s data, and 9 significant microbiome genera from Baxter’s data (*q*-value <0.05). Among them, there were four commonly significant microbiome genera from the two datasets. *Cloacibacillus* was previously found to be related to late-stage CRC patients (Sheng et al., 2019). *Porphyromonas* has been reported to be enriched in gut microbiota profiling of CRC patients in several studies (Yang et al., 2019). *Clostridia vadinBB60 group* was previously found to be enriched in low-graded; right-sided/transverse tumors (Zwinsová et al., 2021). *Streptococcus* was reported to have increased relative abundance in CRA compared to healthy controls (Sun et al., 2020). Furthermore, *Streptococcus gallolyticus* is known as opportunistic pathogen causing infections associated with colon neoplasia in the elderly (Périchon et al., 2022).

## Discussion

In this study, we conducted empirical studies to determine the most appropriate value of *p* combination method for microbiome data. Cauchy combination test was determined to be the most appropriate in terms of type 1 error rates, power, and showed the highest consistency with the true rank than other methods.

The power and type 1 error rates were assessed because it was important to know whether the combined value of *p*s controlled type 1 error rates. For Fisher’s method and Stouffer’s method, the uncontrolled type 1 error rates were observed. Since it was shown that the value of *p*s produced from various methods had significant correlations, Fisher’s method and Stouffer’s method that combine value of *p*s based on the independent assumption of *p*-values tended to show uncontrolled type 1 error rates in some conditions. On the other hand, Kost method incorporating the correlation between the combined *p*-values yielded well-controlled type 1 error rates. Cauchy combination test is a powerful *p*-value combination method robust to arbitrary dependency structures, effectively accounting for the dependency structure of the microbiome dataset.

In our analysis, we considered 12 DE analyses and proposed combining all 12 value of *p*s. Our method can be applicable to any number of DE analyses. For illustrative purposes, we performed the similar analyses using only a fewer DE methods. We considered combining the following methods: (1) taxa-level methods, (2) community-level methods, (3) three randomly chosen methods, (4) five randomly chosen methods, (5) seven randomly chosen methods, (6) a correlated set of methods, (7) another correlated set of methods, and (8) less correlated set of methods. For the randomly chosen three/five/seven methods, we simply applied on a single random set of methods each. Each case resulted similar power trend with that of using all 12 methods (Supplementary Figures 3–10).

In this study, we formulated the difficulty of analyzing microbiome datasets in the sense of overdispersion and high sparsity, by using different analysis methods accounting for these traits. However, one may want to focus on other traits, such as different normalization strategies. We leave it as a future study.

From the rank simulation, Cauchy combination test showed the best performance with significant differences from other value of *p* combination methods for scenarios 1 and 3, while it showed similar performance in scenario 2. Note that scenarios 1 and 3 had six and eight non-causal dependent variables, respectively, while scenario 2 had four non-causal dependent variables and six different causal dependent variables. This implies that Cauchy combination test has the better performance when several non-causal microbiome genera exist. This corresponds to the real microbiome dataset that has several non-causal microbiome taxa and few causal taxa.

The microbiome ranks generated by Cauchy combination test and min P or Simes method did not differ much for the top ranks in the real data analysis. Rather, similar trends of value of *p*s and high correlation coefficients between those methods were observed (Supplementary Figure 11). The difference of microbiome ranks was most obvious with Stouffer’s method, and it was shown that the top ranks generated by Cauchy combination test and Stouffer’s method were quite different. The top ranks generated using Fisher’s method and Kost method did not differ much from those generated using Cauchy combination test. The ranks generated using Fisher’s method and Kost method were the same because they both follow chi-square distributions with different degrees of freedom. Kost method follows a scaled chi-square distribution, but scaling did not alter the resulting ranks.

Most microbiome features have very high sparsity and low abundance, making the statistical analysis difficult. In this study, we considered those characteristics in assessing the different value of *p* combination methods by simulating different levels of sparsity and setting a microbiome feature with high sparsity and low abundance as causal.

The value of *p* combination approach used to determine microbiome importance considering microbiome-specific characteristics can be easily extended to other omics data analyses. For example, our approach can be applied to analysis in RNA-seq or copy number variation data considering data-specific characteristics. There also are several methods to analyze each type of dataset. Note that there is “no one real winner that performs the best.” Thus, combining the results from various methods can have the advantage of using all methods available and being robust to the method-specific assumptions. Cauchy combination test can effectively combine different statistical methods, and produces a representative result of all methods, instead of using a single method that could possibly have a good performance in one dataset, but not in others. Our empirical study showed that the performance of Cauchy combination method provided robust and reasonable result compared to the best performing individual DE method, and performed the best among the value of *p* combination methods in terms of power and rank similarity, and controlling type 1 error rates (supplementary Figure 12). Furthermore, we made a python script with the module “mpmath” that enables floating point arithmetic in case the resulting value of *p*s from individual analysis methods are minute for the combined value of *p* of Cauchy combination test (Cauchy_pval.py). All combination methods used in this script are provided as a R script in https://github.com/HyeonJungHam/P_value_combination, that also includes automatic execution of python script for calculating Cauchy combination test *p*-value.

While Cauchy combination test was introduced with equal weights for each method, it can be easily extended to handle unequal weights. By the authors, Cauchy combination test still accounts for the arbitrary dependency structure when the weights are random variables and independent of test statistics (Liu and Xie, 2020). Thus, it is reasonable to assign a larger weight to the method providing more reliable and accurate result. We expect that the optimal weights would result in an increased performance of Cauchy combination test. However, the choice of optimal weights can change across dataset. Thus, given a dataset, it would not be straightforward to choose the optimal weights. We will leave the choice of optimal weights as a future research topic.

## Data availability statement

Publicly available datasets were analyzed in this study. This data can be found at: The raw sequencing data and metadata of Baxter’s data are available from Sequence Read Archive (SRA) publicly under the accession number of SRP062005. The raw sequencing data and metadata of Zeller’s data are available at European Nucleotide Archive (ENA) with the project number PRJEB6070.

## Ethics statement

Ethical review and approval was not required for the study on human participants in accordance with the local legislation and institutional requirements. The patients/participants provided their written informed consent to participate in this study.

## Author contributions

TP contributed to conception and design of the study, and manuscript revision, read, and approved the submitted version. HH performed the statistical analysis and wrote the first draft of the manuscript. All authors contributed to the article and approved the submitted version.

## Funding

This research was funded by the Bio-Synergy Research Project (2013M3A9C4078158) of the Ministry of Science, ICT and Future Planning through the National Research Foundation, the Korea Health Technology R8D Project through the Korea Health Industry Development Institute (KHIDI), funded by the Ministry of Health and Welfare (HI16C2037).

## Conflict of interest

The authors declare that the research was conducted in the absence of any commercial or financial relationships that could be construed as a potential conflict of interest.

## Publisher’s note

All claims expressed in this article are solely those of the authors and do not necessarily represent those of their affiliated organizations, or those of the publisher, the editors and the reviewers. Any product that may be evaluated in this article, or claim that may be made by its manufacturer, is not guaranteed or endorsed by the publisher.

## Supplementary material

The Supplementary material for this article can be found online at: https://www.frontiersin.org/articles/10.3389/fmicb.2022.990870/full#supplementary-material

## Footnotes

^1^ https://www.r-project.org/
