# Supplementary material for: Combining p-values from various statistical methods for microbiome data
Source: Front Microbiol. 2022 Nov 10;13:990870. doi: 10.3389/fmicb.2022.990870 (PMC9686280; doi:10.3389/fmicb.2022.990870)
Supplement: Supplementary file 1 [file Data_Sheet_1.docx]

**Supplementary Material**

Supplementary Table 1. The microbiome importance by various p-value combination methods. (Baxter’s CRC dataset)

| Taxa (o: order, f:family, g:genus) | Fisher | Min P | Kost | Cauchy | Simes | Stouffer |
| --- | --- | --- | --- | --- | --- | --- |
| o__Rhodospirillales; f__uncultured; g__uncultured | 3 | 1 | 3 | 1 | 1 | 10 |
| o__Veillonellales-Selenomonadales; f__Veillonellaceae; g__Megasphaera | 4 | 2 | 4 | 2 | 2 | 14 |
| o__Gastranaerophilales; f__Gastranaerophilales; g__Gastranaerophilales | 1 | 3 | 1 | 3 | 3 | 1 |
| o__Synergistales; f__Synergistaceae; g__Cloacibacillus | 2 | 4 | 2 | 4 | 4 | 2 |
| o__Bacteroidales; f__Porphyromonadaceae; g__Porphyromonas | 6 | 5 | 6 | 5 | 5 | 9 |
| o__Clostridia_vadinBB60_group; f__Clostridia_vadinBB60_group; g__Clostridia_vadinBB60_group | 5 | 6 | 5 | 6 | 6 | 5 |
| o__Burkholderiales; f__Sutterellaceae; g__Sutterella | 10 | 7 | 10 | 7 | 7 | 18 |
| o__Bacteroidales; f__Marinifilaceae; g__Odoribacter | 11 | 8 | 11 | 8 | 8 | 13 |
| o__Erysipelotrichales; f__Erysipelotrichaceae; g__Turicibacter | 7 | 9 | 7 | 9 | 9 | 3 |
| o__Coriobacteriales; f__Eggerthellaceae; g__Slackia | 8 | 10 | 8 | 10 | 10 | 7 |


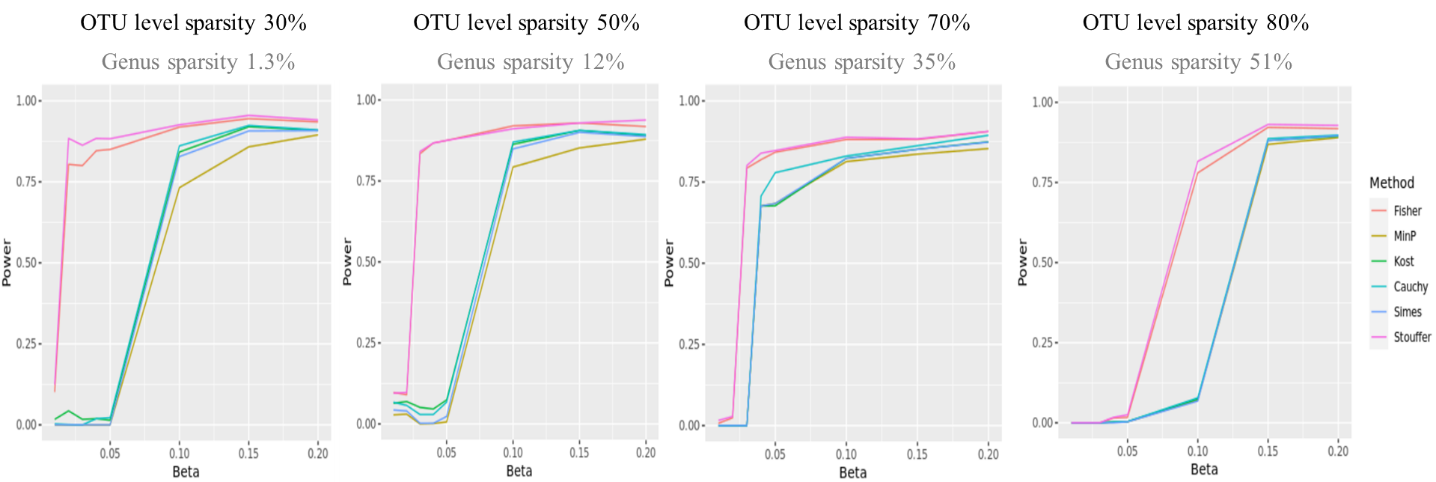


**Supplementary Figure 1. The statistical power of p-value combination methods.** The beta values were given as $\{0.01, 0.02, 0.03, 0.04, 0.05, 0.1, 0.15, 0.2\}$.


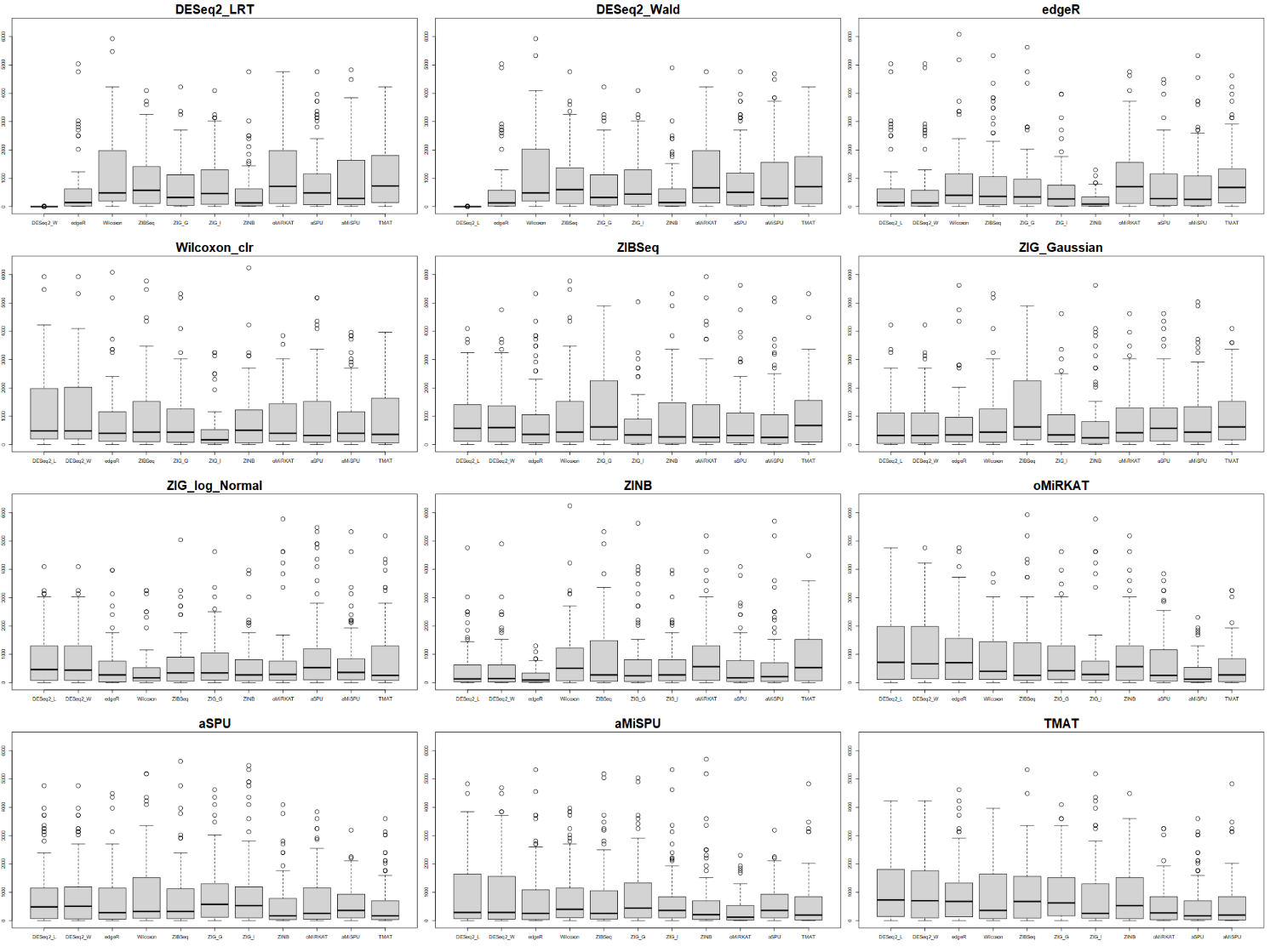


Supplementary Figure 2. The boxplot of rank squared difference with respect to the reference method using Baxter’s CRC dataset. The reference method is written above the boxplot. The methods are from the left DESeq2_LRT, DESeq2_Wald, edgeR, Wilcoxon, ZIBSeq, ZIG_Gaussian, ZIG_log_normal, ZINB, oMiRKAT, aSPU, aMISPU, TMAT without the reference method.


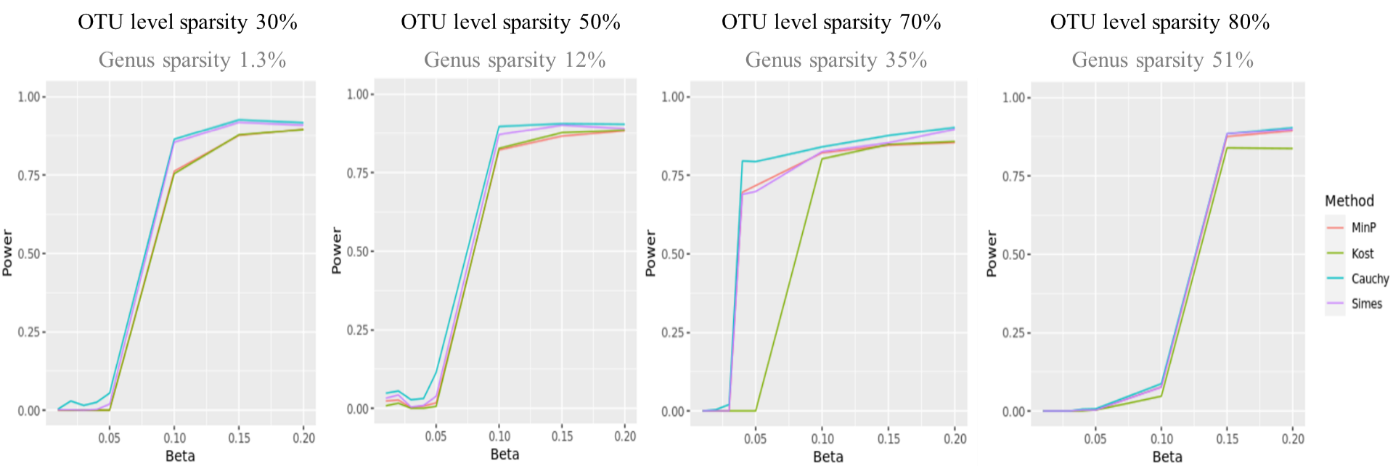


Supplementary Figure 3. The statistical power of p-value combination methods (only taxa-level methods). The beta values are given as {0.01,0.02,0.03,0.04,0.05,0.1,0.15,0.2, 0.5, 0.75, 1.0}.


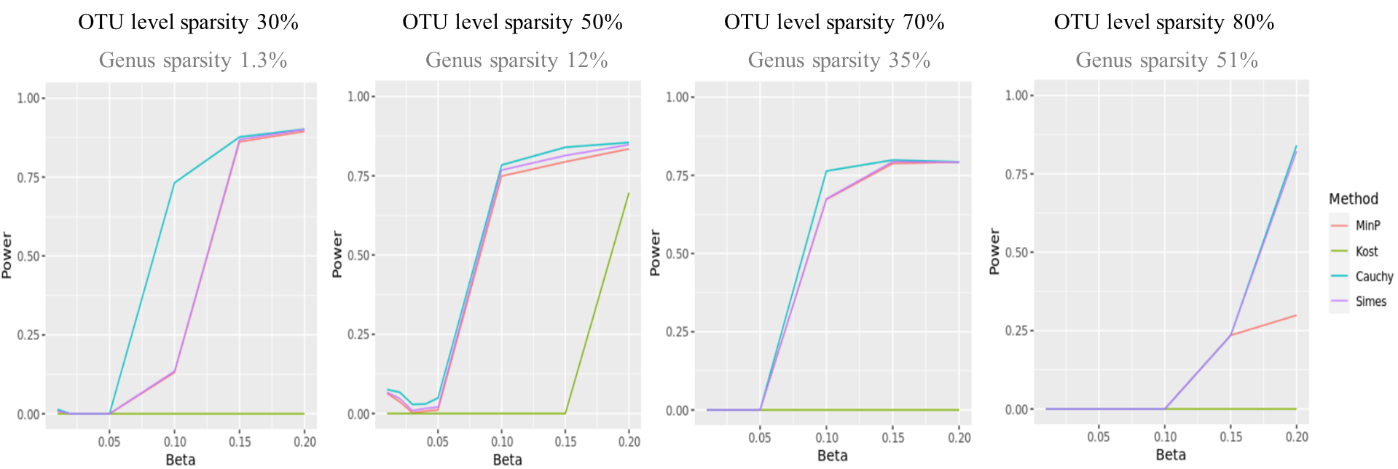


Supplementary Figure 4. The statistical power of p-value combination methods (only community-level methods). The beta values are given as {0.01,0.02,0.03,0.04,0.05,0.1,0.15,0.2, 0.5, 0.75, 1.0}.


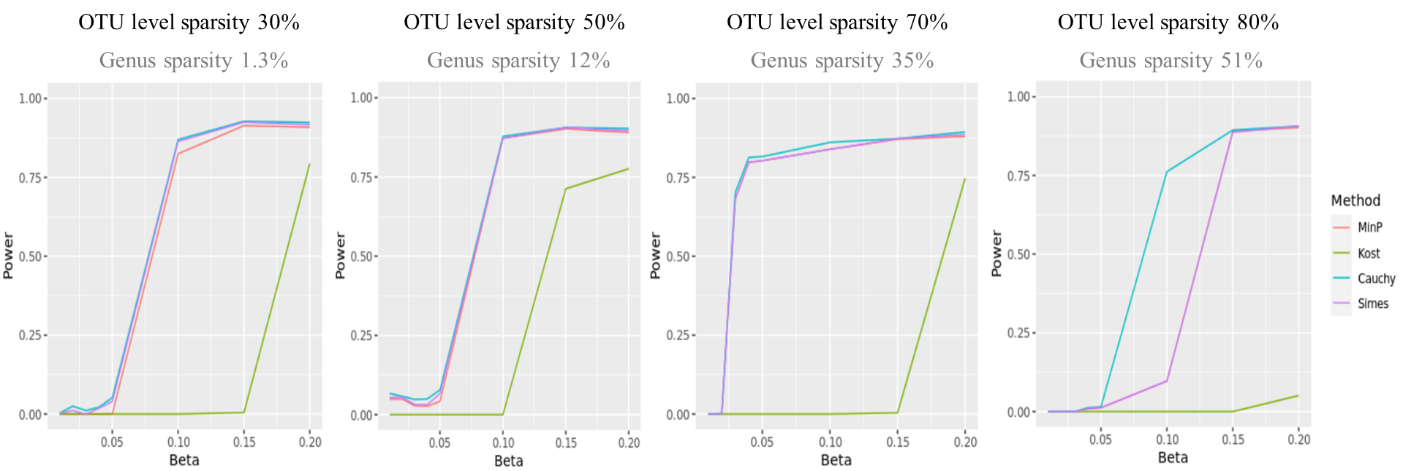


Supplementary Figure 5. The statistical power of p-value combination methods (random 3 methods). The beta values are given as {0.01,0.02,0.03,0.04,0.05,0.1,0.15,0.2, 0.5, 0.75, 1.0}.


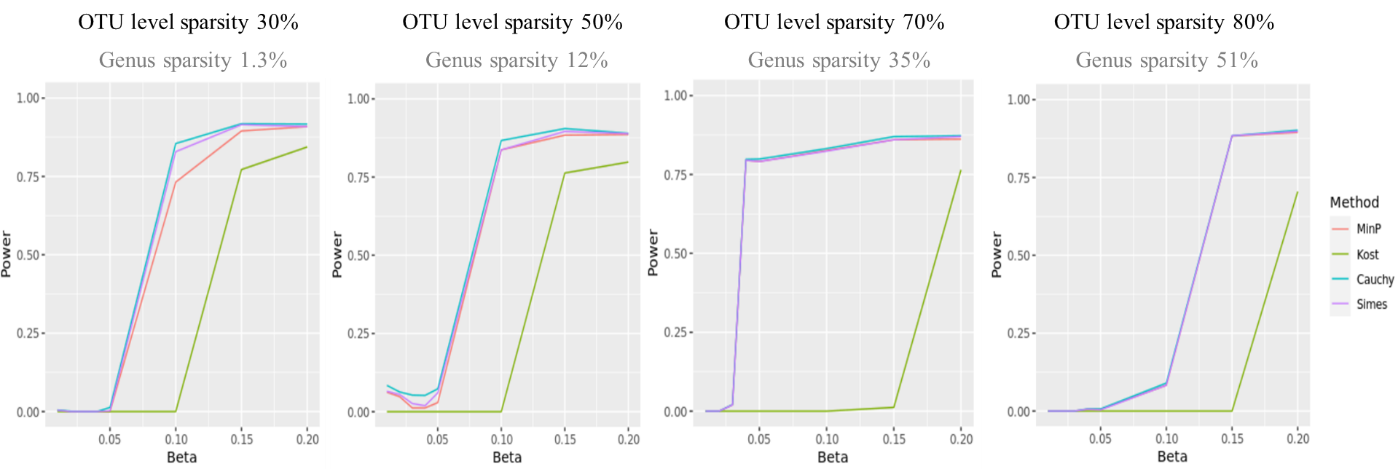


Supplementary Figure 6. The statistical power of p-value combination methods (random 5 methods). The beta values are given as {0.01,0.02,0.03,0.04,0.05,0.1,0.15,0.2, 0.5, 0.75, 1.0}.


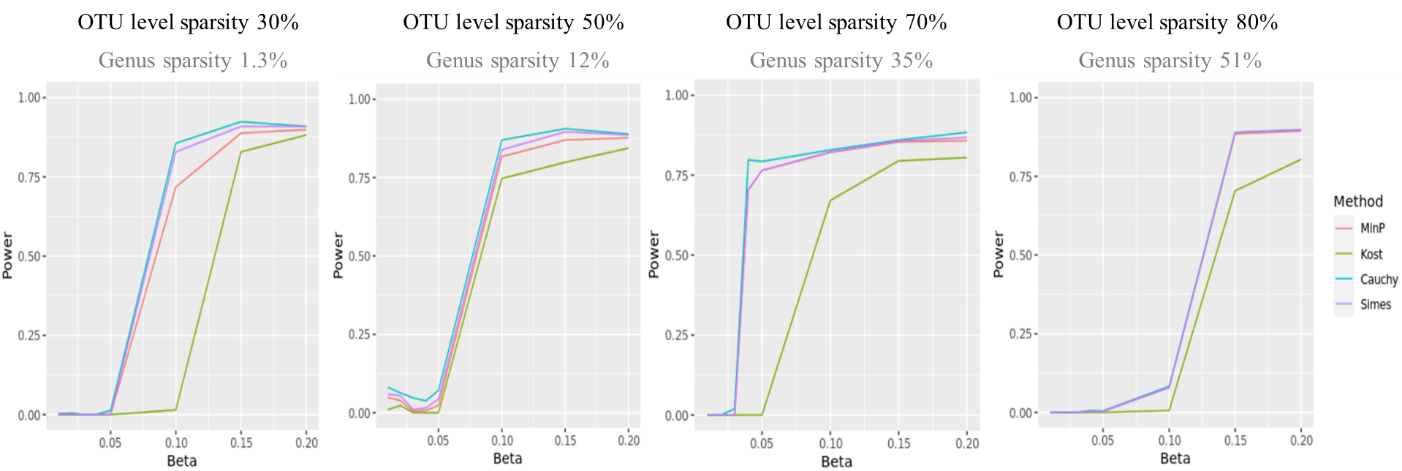


Supplementary Figure 7. The statistical power of p-value combination methods (random 7 methods). The beta values are given as {0.01,0.02,0.03,0.04,0.05,0.1,0.15,0.2, 0.5, 0.75, 1.0}.


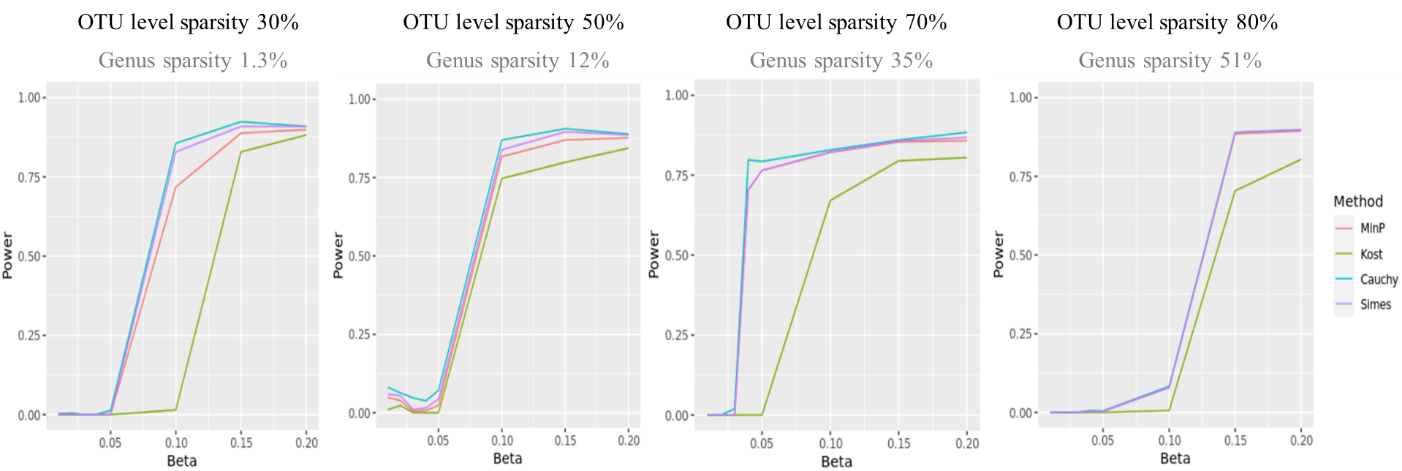


Supplementary Figure 8. The statistical power of p-value combination methods (a correlated set of methods, DESe q2, edgeR, and ZINB). The beta values are given as {0.01,0.02,0.03,0.04,0.05,0.1,0.15,0.2, 0.5, 0.75, 1.0}.


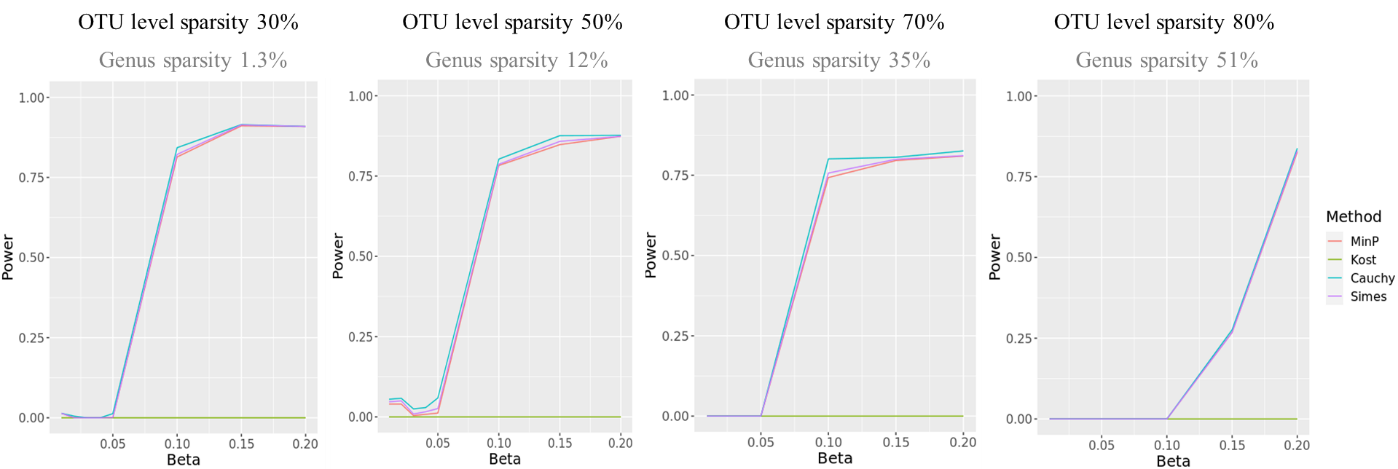


Supplementary Figure 9. The statistical power of p-value combination methods (another correlated set of methods, ZIBSeq, oMiRKAT, aSPU). The beta values are given as {0.01,0.02,0.03,0.04,0.05,0.1,0.15,0.2, 0.5, 0.75, 1.0}.


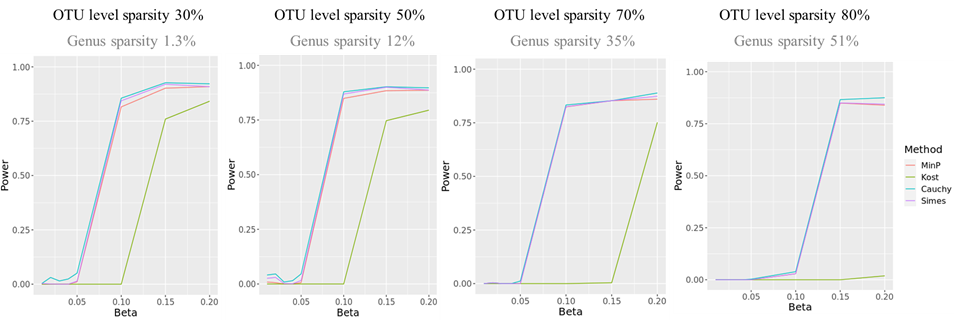


Supplementary Figure 10. The statistical power of p-value combination methods (less correlated set of methods, DESeq2_LRT, Wilcoxon_CLR, ZIBseq, TMAT). The beta values are given as {0.01,0.02,0.03,0.04,0.05,0.1,0.15,0.2, 0.5, 0.75, 1.0}.


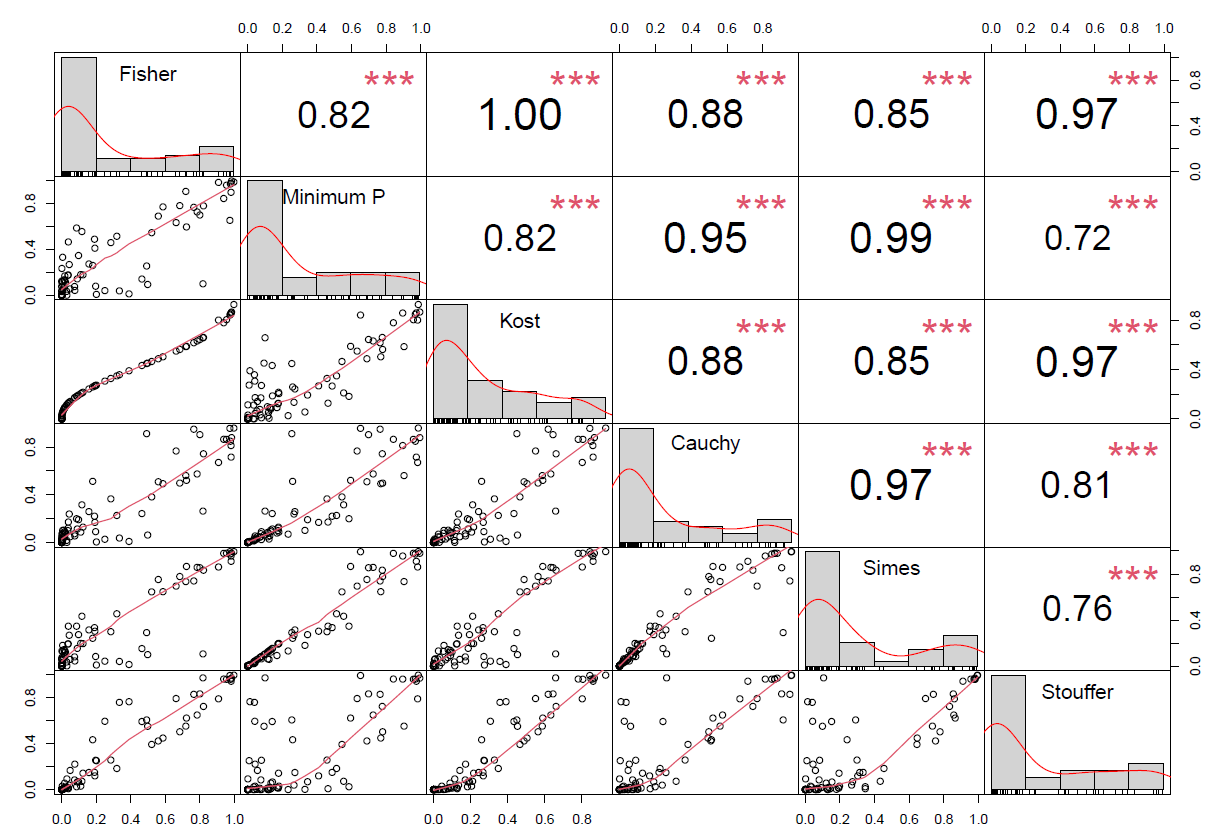


Supplementary Figure 11. Pairwise Spearman rank correlation coefficient between p-value combination methods in the Baxter’s CRC dataset.


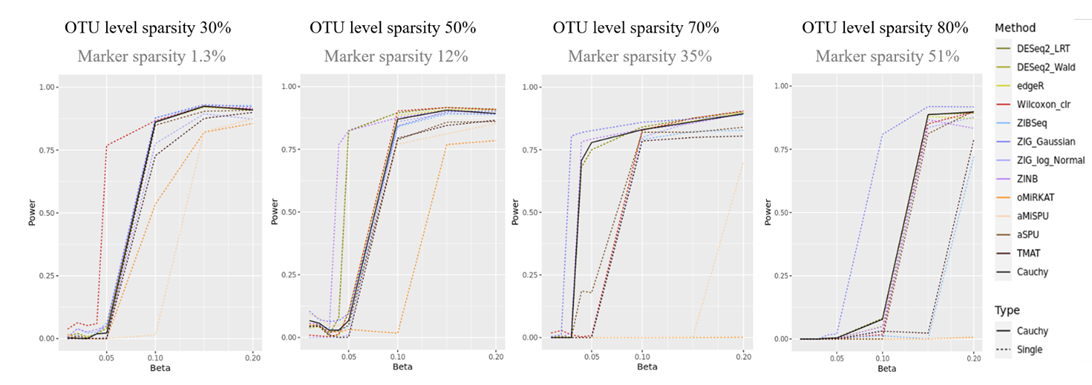


Supplementary Figure 12. The statistical power of p-value combination methods. The x-axis represents the degree of association ($\boldsymbol{\beta}$). The value of $\boldsymbol{\beta s}$ were given as $\boldsymbol{\{0.01, 0.02, 0.03, 0.04, 0.05, 0.1, 0.15, 0.2\}}$. The y-axis represents the power.
